# Risk factors for SARS-CoV-2 transmission in student residences: a case-ascertained study

**DOI:** 10.1186/s13690-022-00966-4

**Published:** 2022-09-21

**Authors:** Marte Vanbesien, Geert Molenberghs, Caspar Geenen, Jonathan Thibaut, Sarah Gorissen, Emmanuel André, Joren Raymenants

**Affiliations:** 1 Faculty of Medicine, KU Leuven, Herestraat 49, 3000 Leuven, Belgium; 2 Interuniversity Institute for Biostatistics and Statistical Bioinformatics, Data Science Institute, Hasselt University, Martelarenlaan 42, 3500 Hasselt, Belgium; 3 Interuniversity Institute for Biostatistics and Statistical Bioinformatics, KU Leuven, Kapucijnenvoer 35, 3000 Leuven, Belgium; 4 Department of Microbiology, Immunology and Transplantation, KU Leuven, Herestraat 49, 3000 Leuven, Belgium; 5 Department of Laboratory Medicine, University Hospitals Leuven, Herestraat 49, 3000 Leuven, Belgium

**Keywords:** COVID-19, SARS-CoV-2, Transmission, Risk factors, Student residence, Shared household, Congregate setting

## Abstract

**Background:**

We aimed to investigate the overall secondary attack rates (SAR) of COVID-19 in student residences and to identify risk factors for higher transmission.

**Methods:**

We retrospectively analysed the SAR in living units of student residences which were screened in Leuven (Belgium) following the detection of a COVID-19 case. Students were followed up in the framework of a routine testing and tracing follow-up system. We considered residence outbreaks followed up between October 30th 2020 and May 25th 2021. We used generalized estimating equations (GEE) to evaluate the impact of delay to follow-up, shared kitchen or sanitary facilities, the presence of a known external infection source and the recent occurrence of a social gathering. We used a generalized linear mixed model (GLMM) for validation.

**Results:**

We included 165 student residences, representing 200 residence units (N screened residents = 2324). Secondary transmission occurred in 68 units which corresponded to 176 secondary cases. The overall observed SAR was 8.2%. In the GEE model, shared sanitary facilities (*p* = 0.04) and the recent occurrence of a social gathering (*p* = 0.003) were associated with a significant increase in SAR in a living unit, which was estimated at 3% (95%CI 1.5-5.2) in the absence of any risk factor and 13% (95%CI 11.4-15.8) in the presence of both. The GLMM confirmed these findings.

**Conclusions:**

Shared sanitary facilities and the occurrence of social gatherings increase the risk of COVID-19 transmission and should be considered when screening and implementing preventive measures.

**Supplementary Information:**

The online version contains supplementary material available at 10.1186/s13690-022-00966-4.

## Background

The COVID-19 pandemic has caused over 6.3 million reported deaths as of June 2022 [1]. Despite the buildup of natural and vaccine-based immunity, widespread community transmission of COVID-19 continues to put pressure on health systems. Non-pharmaceutical interventions including isolation of confirmed cases, tracing and quarantining of contacts, and testing of symptomatic and at-risk individuals, may therefore remain needed to mitigate the overall impact [2].

Congregate settings, such as curative and residential care settings, prisons, and student residences, are at risk of rapid COVID-19 transmission due to crowding and frequent close contact [3]. They are suggested to start outbreaks that spill over to other high-risk settings and the community [4]. Students living in residences pose additional risk to the community due to high contact rates in this age cohort [5].

Despite these risks, there is a paucity of data on the range of secondary attack rates (SAR) one may find in student residences, which risk factors underpin the observed variation in transmission and how they compare to regular households [6–8]. Some studies suggest a higher risk of transmission if residents share living spaces [9] or if they do not adhere to prevention measures [8]. However, the sample size in these studies is too small to draw any reliable conclusions. Studies examining COVID-19 transmission in the household setting identified the number of household contacts, the nature of relationship between contacts, the age of contacts and the presence of symptoms as risk factors for higher rates of secondary transmission [10], but even in the household setting, there is only limited data about the influence of behaviour- or infrastructure-related factors on the secondary infection probability. One study observed a trend towards higher SAR in households if members kissed, hugged, shared sanitary facilities or shared a bed, although these results were not found to be significant [11]. Furthermore, it is unclear whether these supposed risk factors in household settings are transferable to other high-risk settings such as student residences. This lack of evidence base limits the implementation of effective preventive measures and a comprehensive testing strategy which balances effectiveness with proportionality.

To fill this gap, we conducted a retrospective case-ascertained study and analyzed 165 instances in which a student residence was screened in the student city of Leuven, Belgium, following the detection of at least one case of COVID-19 in the residence. We quantified the SAR and collected information related to the living arrangements and interactions within and outside the screened residences to evaluate whether they were associated with a higher SAR.

## Materials and methods

### Setting and design

**Fig. 1:**
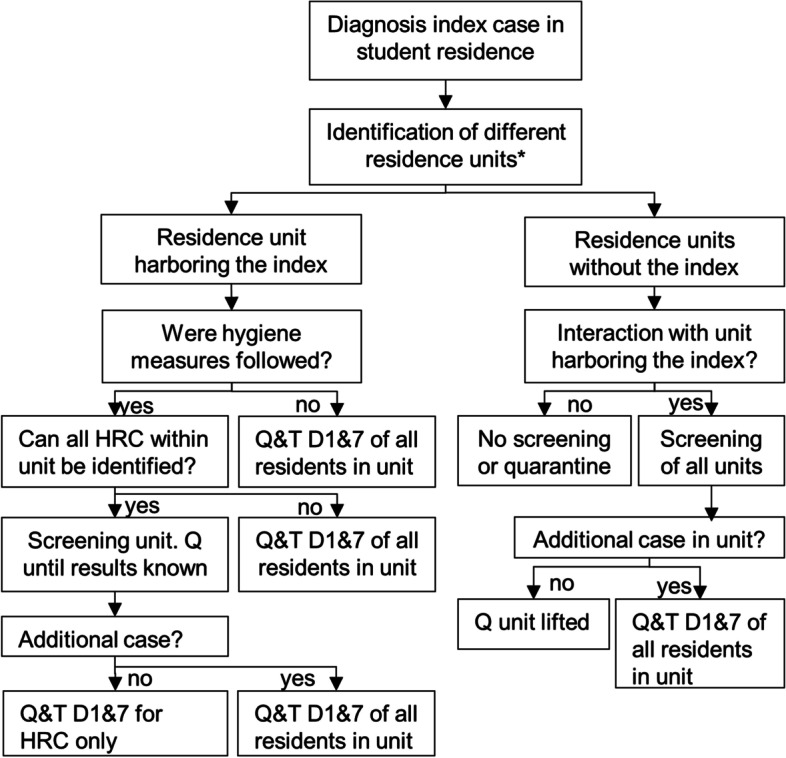
Screening algorithm during a possible student residence outbreak. Abbreviations: D1 = day one, as soon as possible after the diagnosis of the index case. D7 = Day seven, seventh day after the day of diagnosis of the index case, Q = Quarantine, T = testing, HRC = high-risk contact. *Residence unit: students sharing a kitchen or sanitary facilities. Screening was as follows: if an index case recently resided in a student residence, all students who structurally/contractually shared either the same kitchen or sanitary facilities with them and who had also resided in the residence in the week leading up to the onset of symptoms or diagnosis of the index, were invited for testing as soon as possible. They were part of the same residence unit. Contacts who were already diagnosed with COVID-19 between 14 and 60 days prior to the index were not eligible for screening. A subset of students in this residence unit was additionally asked to quarantine and undergo a second test on day 7. This subset depended on whether hygiene measures were strictly complied with and whether high-risk contacts (contact for > 15′ at < 1,5 m without face masks, or direct physical contact) could be readily identified through contact tracing. If other living units interacted regularly with the one harboring the initial index, those other units were also invited for a first test. In case additional cases were diagnosed in a particular unit, students belonging to this unit were asked to quarantine and undergo a second test on day seven. The detection of new cases in a unit could lead to additional screening rounds following the same protocol

**Fig. 2:**
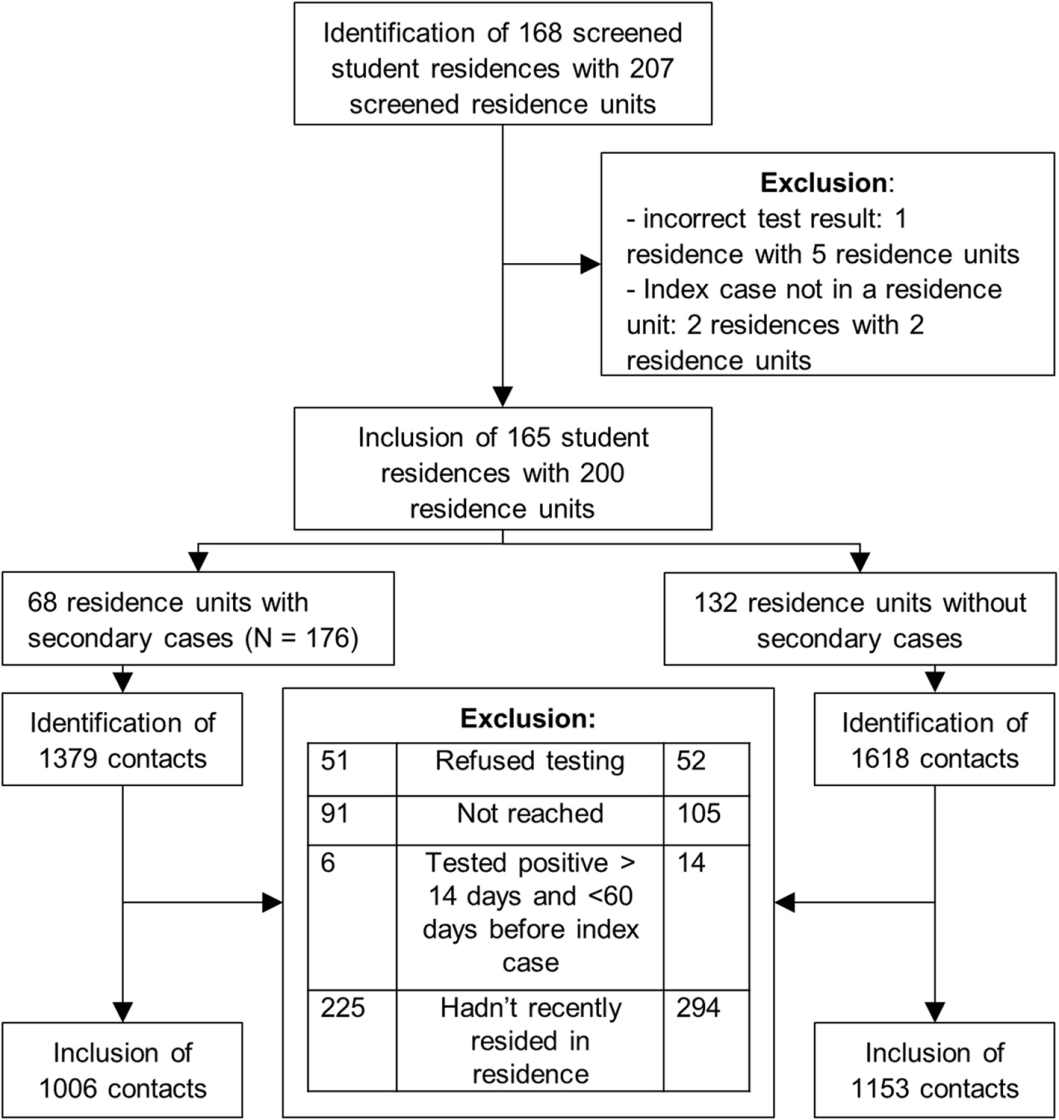
Inclusion and exclusion of student residences and contacts during the study period*.* We included all students residing in the same residence as a newly diagnosed index case as contacts if they met the above criteria for further testing. We excluded contacts who were lost to follow up and who were already diagnosed with COVID-19 between 14 and 60 days before the diagnosis of the index. We also excluded contacts that had not resided in their residence in the week leading up to the onset of symptoms or diagnosis of the first case

This retrospective case-ascertained study was performed on data gathered in the context of a testing and contact tracing system targeted to over 30.000 tertiary education students in Leuven, Belgium [12, 13]. A standard screening protocol for student residences was introduced on October 30th 2020 and aimed to strike a balance between effectiveness and proportionality (Fig. 1). All data from cases and contacts gathered during the follow-up of a residence outbreak were coded into a customized version of Go.Data. The inclusion and exclusion criteria for residences, residence units and individuals are described in Fig. 2. A student residence was defined as an architectural complex housing mostly tertiary education students. A residence unit was defined as a group of student rooms within a residence that shared either sanitary or kitchen facilities.

### Outcome variables

For each student residence, we labelled the case who was first diagnosed with COVID-19 as the index case. The selection and inclusion of contacts is shown in Figs. 1 and 2. Contacts were labelled positive if they were diagnosed with COVID-19 in the next 2 weeks and negative if they did not test positive and underwent at least one negative PCR or antigen test in the 2 weeks following their last contact with the index case or the residence. The SAR in a residence unit was defined as the number of secondary cases in the unit divided by the total number of contacts from the unit tested.

### Covariates

We examined the impact of the following covariates on the SAR in a residence unit:

- The delay between onset of symptoms in the index case and screening of the residence: < 4 days delay, ≥ 4 days.
- Whether or not the index case shared a kitchen with others.
- Whether or not the index case shared sanitary facilities with others.
- Whether or not the index reported being infected by a source external to the residence. If the index tested positive during quarantine after traveling abroad, traveling was considered the external source.
- Whether or not the index attended a social gathering in the residence in the 7 days prior to onset of symptoms or diagnosis. The gathering was characterized by at least two of the following: crowding (at least five individuals belonging to at least two different households), close contact (< 1.5 m, without the use of face masks) and closed environment (indoor).

In a sensitivity analysis focusing only on the residence units harboring the index case, we distinguished both types of units by adding the binary parameter ‘index case present in the residence unit’.

If two students residing in the same residence tested positive on the same day, both were considered index cases. One of both was counted as a secondary infection when determining the SAR. An ‘OR’ logic was used for determining the labels of the covariates, meaning it sufficed if one of both index cases reported the presence of the covariate to classify the variable as ‘present’. When the 2 index cases were part of a different residence unit, both units were classified as units with an index case present.

### Statistical analysis

We analyzed the impact of our covariates on the secondary attack rate by means of logistic regression while correcting for correlation within the residence unit and residence. We used generalized estimating equations (GEE) for our primary analysis, which describes the average SAR one may expect when screening a residence in the presence of certain covariate levels. A generalized linear mixed model (GLMM) was used as a validation method. GLMM allows for a random effect and therefore corresponds to the full range of SAR one can encounter in an individual residence unit. Backward elimination was used to establish a model in which only significant effects remained. We performed a sensitivity analysis looking only at the units harboring the initial index case in the residence. Detailed information on the statistical methods used can be found in Supplementary materials.

## Results

### Included participants

Of the 168 student residences screened in Leuven or boroughs between October 30th 2020 and May 25th 2021 following the detection of at least one confirmed case, 165 residences were included. They represented 207 residence units. Three residences (7 units) were excluded as the index reported not being part of a residence unit or had an incorrect test result. In seven instances, two students residing in the same residence tested positive on the same day. Both were thus considered index cases. This brings the total of identified index cases to 172. We identified 2997 contacts meeting the criteria for screening. We excluded a total of 838 because: they refused testing (*n* = 103), could not be reached by the contact tracing team (*n* = 196), were not recently present in the student residence (*n* = 519) or had had a COVID-19 infection between 14 and 60 days prior (*n* = 20). This left 2159 contacts for inclusion in the analysis (Fig. 2), with seven of them corresponding to duplicate index cases. This makes a total of 2324 tested individuals (172 index cases + 2159 contacts – 7 (counted both as index and contact)). See Supplementary Fig. 1 in Additional file 1 for the distribution of outbreaks over time.

### Student residence characteristics

The main characteristics of the residence units are presented in Table 1. Secondary transmission occurred in 68/200 residence units. This corresponded to 176 secondary cases. The median number of secondary cases was 2 per unit (IQR 1-3). The number of negatively screened students ranged between 1 and 103 per residence unit with a median of 7.5 (IQR 5-11). The overall observed secondary attack rate was consequently 8.2% (176/2159). Symptoms were present in the index in 77% (154/200) of units. The delay time between the onset of symptoms and the follow-up of the residence was shorter than 4 days in 44.5% (89/200) and longer than 4 days in 29.5% (59/200). In 83.8% (170/200) residence units, the index case shared a kitchen with other students, most frequently with between 6 and 10 others. The index case also shared sanitary facilities in 131/200 (65.5%) of the residence units, again most frequently with 6 to 10 others. In about half of the residence units, the index case reported a possible source of infection outside the student residence. In half of the residence units, the index case reported having attended a social gathering in the student residence in the week before their onset of symptoms or diagnosis. In 83.5% (167/200) of included units, the index case who was first diagnosed in the residence, resided in that unit. This means that 16.5% (33/200) of residence units were screened because of their interaction with a unit harboring the first index case in the residence. No demographics were collected on index cases or contacts. However, the mean age of all students tested in the university’s test center during the study period was 23 years old.

**Table 1:** Characteristics of residence units

	Total (n(%)) (***n*** = 200)	Residence units with secondary cases (n(%)) (***n*** = 68)	Residence units without secondary cases (n (%)) (***n*** = 132)
Symptoms of index case	–	–	–
Present	154 (77)	57 (83.8)	97 (73.5)
Not present	34 (17.0)	4 (58.8)	30 (22.7)
Not reported	12 (6.0)	7 (10.3)	5 (3.8)
Shared kitchen	–	–	–
No	14 (7)	4 (5.9)	10 (7.6)
≤ 5 students	29 (14.5)	8 (11.8)	21 (15.9)
6-10 students	60 (30.0)	21 (30.9)	39 (29.5)
11-15 students	44 (22)	13 (19.1)	31 (23.5)
16-20 students	19 (7.8)	10 (14.7)	9 (6.8)
> 20 students	18 (9.5)	10 (14.7)	8 (6.0)
Not reported	16 (8.0)	2 (2.9)	14 (10.6)
Shared sanitary facilities	–	–	–
No	52 (26.0)	14 (20.6)	38 (28.8)
≤ 5 students	43 (21.5)	16 (23.5)	27 (20.5)
6-10 students	52 (26.0)	17 (25.0)	35 (26.5)
11-15 students	22 (11)	9 (13.2)	13 (9.8)
16-20 students	9 (4.5)	5 (7.4)	4 (3.0)
> 20 students	5 (2.5)	4 (5.9)	1 (0.8)
Not reported	17 (8.5)	3 (4.4)	14 (10.6)
External infection source	–	–	–
Yes	107 (53.5)	32 (47.1)	75 (56.8)
No	39 (19.5)	15 (22.1)	24 (18.2)
Not reported	54 (27.0)	21 (30.9)	33 (25.0)
Social gathering	–	–	–
Yes	100 (50.5)	48 (70.6)	52 (39.4)
No	49 (24.5)	10 (14.7)	39 (29.5)
Not reported	51 (25.5)	10 (14.7)	41 (33.1)
Index case present	–	–	–
Yes	167 (83.5)	58 (85.3)	109 (82.6)
No	33 (16.5)	10 (14.7)	23 (17.4)

Clarifications & abbreviations: Shared kitchen: index case shared a kitchen with others. Shared sanitary facilities: index case shared sanitary facilities with others. External infection source: index case reported a possible external infection source. Social gatherings: The recent occurrence of at least one social gathering in the student residence attended by the index case. Index case present: the residence unity harbored the first index case diagnosed in the residence

*n* number of residence units

### GEE on all residence units

**Fig. 3:**
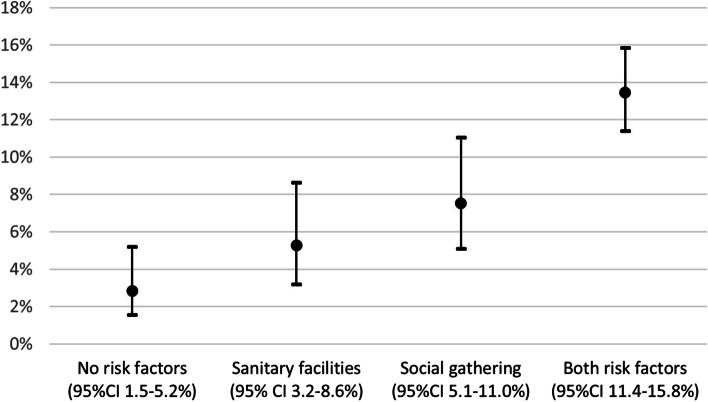
SAR, estimated by GEE based on the absence or presence of significant risk factors. Abbreviations: CI = confidence interval, SAR = secondary attack rate. The SAR increased by 10.6% when moving from the units without any risk factor to the units with both risk factors present

Using a GEE model, the overall SAR within a living unit was estimated to 8.1% (95%CI 7.1-9.4%). Of the 5 covariates assessed, 3 were removed through backward elimination because they were not found to significantly influence secondary transmission: the delay between symptom onset in the index case and screening of the residence, the shared use of a kitchen and the fact that they had a known source outside of the residence. This left the shared use of sanitary facilities (*p* = 0.04) and the occurrence of a social gathering in the student residence attended by the index case (*p* = 0.003) as the statistically significant predictors of secondary transmission. The SAR was lowest at 3% (95%CI 1.5-5.2%) for the residence units without either of the risk factors. It was highest at 13% (95%CI 11.4-15.8) when both risk factors were present. An interim position was occupied by units with one risk factor (Fig. 3).

To validate these findings, a GEE model was built using the subset of residence units harboring the initial index case. The same covariates were found to be significant. They had a similar impact on estimated SAR (Additional file 1 - SI statistical analysis).

### GLMM on all residence units

**Fig. 4:**
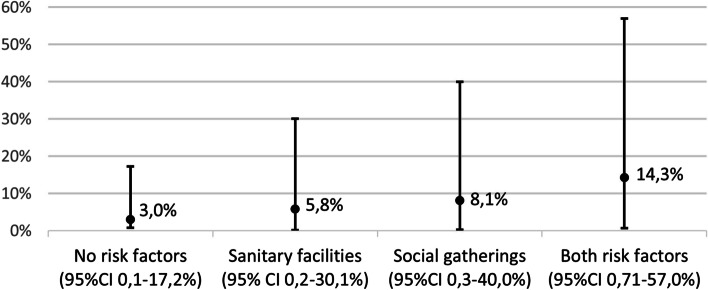
SAR, estimated by GLMM based on the absence or presence of significant risk factors. Abbreviations: CI = confidence interval, sanitary facilities: the index case shared sanitary facilities with others, social gatherings: the occurrence of a social gathering in the residence, which was attended by the index case. The SARs had a large 95% confidence interval since GLMM allows for a random effect and therefore corresponds to the full range of expected SAR one can encounter in an individual residence unit

Using a GLMM model, a similar secondary attack rate was found: 8.6% (95% CI 0-47%). Its 95% CI was much wider as it allows for a random effect and therefore corresponds to the full range of expected SAR one can encounter in an individual residence unit. The occurrence of a social gathering (*p* = 0.002) remained a significant risk factor. Sharing sanitary facilities was borderline non-significant in this model (*p* = 0.088). However, it was retained for coherence between both approaches. The impact of these covariates on the SAR was similar to the GEE model (Fig. 4). A GLMM model including only residence units harboring the initial index case showed similar covariate weights and SAR (SI statistical analysis).

## Discussion

In this large case-ascertainment study, we examined the SAR of COVID-19 infections in a cohort of 165 student residences representing 200 living units and 2324 tested individuals. Standardized risk-based screening protocol was used in all instances following the diagnosis of a first index case in the residence.

Our results show that the overall SAR in student residence units is estimated to be around 8%, which is lower than the SAR observed in Belgian households during a similar study period [14] and other household transmission studies during the alpha dominant pre-vaccination era [15, 16]. The results are in line with the 7.8% SAR, observed in student residences in a study taking place in a similar setting in the United Kingdom [17]. A second study, which investigated COVID-19 transmission in group living environments in Japan, examined three student dormitories and reported a much higher average secondary attack rate of 27.5%. This study only included three student dormitories. Just one had secondary infections, with a SAR of 57%. Furthermore, the outbreak had likely originated from a high-risk event outside the dormitory [7].

Additionally, we evaluated the influence of 5 risk factors on the overall SAR observed when screening a residence unit, two of which were found to significantly influence the risk of transmission, leading to a SAR that ranged from 3% (95%CI 1.5-5.2) in the absence of either risk factor to 13% (95%CI 11.4-15.8) in the presence of both as assessed by a generalized estimating equations model.

First, the occurrence of an indoor crowded social gathering in the student residence attended by the index case was observed to increase onward transmission, corroborating previous findings pointing at the risk of attending high-risk indoor activities [11, 18, 19] and pointing out the importance of indoor social events characterized by crowding and close contact in sparking onward transmission.

Second, the shared use of sanitary facilities significantly increased the probability of identifying secondary cases during screening. This association was found to be a significant factor for acquiring COVID-19 in univariate analysis in one other study. While sanitary facilities were shared in 74% of residence units, preventive measures dissuaded students from concurrent use during the study period. As separations generally exist between installations, this association cannot be explained by droplet transmission. This leaves aerosol-transmission, indirect fomite transmission, fecal-oral and fecal-aerosol transmission as possible underlying mechanisms [20–22]. Alternatively, it could imply that shared use of sanitary facilities constitutes a proxy for overall exposure to an index case living in the same residence unit.

Three factors were not found to be significant, namely whether the index case shared a kitchen, whether they had a known source of infection outside of the residence and whether the delay between onset of symptoms in the index case and the screening of the residence was < 4 days or ≥ 4 days. For any of these factors, a lack of power may be at play. With regards to the sharing of a kitchen, the fact that a larger group of individuals generally shared a kitchen than a bathroom (Table 1) may imply that the overall exposure to other residents using the same kitchen is generally low. Alternatively, building characteristics or preventive measures in place during the study period may have played a role as well. The fact that the presence of a clear external source of infection in the index case does not significantly influence the SAR within the residence implies that this criterion cannot be used to abstain from screening the residence after diagnosis of a case of COVID-19. While a long delay between symptom onset in the index case and the screening of the unit was not significant, the *p*-value of 0.0691 in the initial multivariate GEE model for all residence units does convey a trend (see SI in Additional file 1).

Sensitivity analyses restricted to residence units harboring the initial index case show that the presence or absence of the initial index case in a residence unit had no significant impact on our conclusions. However, only 17% of the included residence units did not harbor the initial index case and thus the proportion might be too small to draw any firm conclusions. Additionally, residence units which did not harbor the initial index case were only screened and included in this study if there was reported interaction with the residence unit harboring the initial index case. While our analysis corrected for correlation within the same outbreak, further research assessing infection probability in all residence units in a student residence, regardless of interaction between units, can better elucidate the full impact of this parameter.

Our results provide valuable insights into how risk factors assessed in the first index case of COVID-19 in a student residence can inform the subset of residents to be screened thereafter. The crude SAR we observed was high, at about 8%, even though our screening protocol was much broader than screening only contacts with a direct exposure to the first index case. The risk factors found to significantly influence the SAR in the current study provide an evidence base for informed decision making on which subset of individuals should undergo screening in a student residence, thereby improving the balance between comprehensiveness and proportionality of the deployed strategy. Our GLMM analysis demonstrates, however, that a large variation of SAR can still be found even when taking into consideration the risk factors identified.

Also, our results improve the evidence base for implementing preventive measures. The importance of shared sanitary facilities is – in the light of the scant and circumstantial evidence base for fecal-oral transmission – rather suggestive of the importance of ventilation for limiting transmission. This fact is also underscored by the increased SAR we observed if a social event had taken place at the residence prior to diagnosis of the first case.

As student residences have many characteristics in common with other collective households, our results have implications for the screening and prevention measures in curative and residential care settings, prisons and the like.

Finally, the fact that social events seem to spur onward transmission implies the need for broader screening of attendants of events characterized by crowding, close contact and closed environment regardless of where this venue may have taken place.

## Limitations

Our study has several limitations. First, the self-reported nature of most of our data may be subject to recall and reporting bias. Second, the population we examined was almost entirely unvaccinated and we did not consider natural immunity in cases or contacts. Third, the alpha variant-of-concern was the dominant variant involved in most of the outbreaks. Fourth, general contact restrictions varied throughout the study (SI fig. 2 in Additional file 1) [23]. Fifth, additional parameters likely to influence transmission, such as compliance with preventive measures, university holidays and detailed building characteristics, were not assessed. Sixth, our analyses were performed on a residence unit level, but not all analyzed parameters were equal for all students in one residence unit. Further research assessing the risk of infection in contacts at the personal level is thus warranted. Lastly, the power of this study may not have been sufficient to detect an impact from epidemiologically relevant risk factors. Larger studies may be required to discern them.

## Conclusions

We investigated the association of site- and behavior-specific characteristics on the secondary attack rate of COVID-19 in student residences. Each of these can be elucidated before screening. The large sample of screened residences and the predefined and rigorous screening algorithm allowed for an accurate assessment of the SAR. Additionally, our models show that both indoor crowded social gatherings and shared sanitary facilities were independent risk factors for transmission. While the presence or absence of these risk factors does not explain all variability in the SAR, they are still important to consider when designing preventive measures, an efficient screening algorithm and student residences and other shared households. No other study in student residences has found these risk factors to be independently associated with SARS-CoV-2 transmission. The power of our study may have been too limited to discern additional epidemiologically significant risk factors.

## Supplementary Information

**Additional file 1: S1.** Statistical analysis – secondary attack rate. **Supplementary 2.** Sensitivity analysis. **Supplementary Figure 1.** Timing of outbreaks in student residences. **Supplementary Figure 2.** Google mobility data between October 30th 2020 and May 25st 2021 for the region of Flemish Brabant, Belgium.

**Additional file 2.**

## Data Availability

The data underlying this article are available in the article and in its online Supplementary material.

## Abbreviations

GEE: Generalized estimating equations
GLMM: Generalized linear mixed model
SAR: Secondary attack rate
